# Multisite Mini-Implant Anchorage for Maxillary Intrusion With Clear Aligners: A Finite Element Analysis

**DOI:** 10.1016/j.identj.2026.109728

**Published:** 2026-07-15

**Authors:** Shengyou Chen, Shaochuan Feng, Wangxi Ni, Yutao Pei, Xiaoyan Wang, Xinze Zhang

**Affiliations:** a School of Mechanical Engineering, University of Science and Technology Beijing, Beijing, China; b Department of Stomatology, Beijing Tiantan Hospital, Capital Medical University, Beijing, China; c Department of Advanced Production Engineering, Engineering and Technology Institute Groningen, Faculty of Science and Engineering, University of Groningen, Groningen, The Netherlands; d Department of Civil Engineering, Tsinghua University, Beijing, China

**Keywords:** Clear aligner, Mini-implant, Full-arch intrusion, Finite element analysis, Anchorage design, Vertical control

## Abstract

**Objective:**

To investigate the biomechanical effects of various mini-implant anchorage configurations on maxillary full-arch intrusion with clear aligners and to propose an optimized protocol for vertical control in clinical orthodontics.

**Methods:**

Based on cone-beam computed tomography data from an adult patient with Angle Class II high-angle malocclusion, three-dimensional finite element models of the maxillary arch, periodontal ligament (PDL), maxilla, clear aligner, mini-implants, and traction buttons were constructed. Simulations were performed for three anchorage categories: (1) intrusion without auxiliary anchorage (control group); (2) intrusion with posterior-only anchorage, comprising three groups – buccal-only, palatal-only, and bilateral – with mini-implants placed between the second premolar and first molar (#5/#6); and (3) intrusion with combined anterior-posterior anchorage, including a central incisor-posterior anchorage group (five implants) and a lateral incisor-posterior anchorage group (six implants). Outcomes included tooth displacement and PDL hydrostatic pressure distribution.

**Results:**

Among the control group and the three posterior anchorage groups, bilateral posterior anchorage produced the most bodily posterior intrusion. Lateral incisor–posterior anchorage achieved the most optimal intrusion of the arch and mitigated the sagittal inclination of the anterior teeth. Posterior teeth in the second quadrant exhibited significantly smaller displacement and PDL hydrostatic pressure than those in the first quadrant, attributable to the fused, significantly thicker root morphology of the second-quadrant second molar (#27).

**Conclusion:**

Bilateral posterior anchorage effectively balances the buccal and palatal moments and is recommended for posterior intrusion. Strategic placement of mini-implants in the anterior region can prevent anterior tooth inclination and facilitate posterior intrusion, thereby achieving coordinated vertical control, although this approach carries an increased risk of root resorption. Individual anatomical features, particularly root morphology, should be considered in clinical vertical control protocols.

## Introduction

Since its introduction by Align Technology in 1997, Clear Aligner Therapy (CAT) has gained widespread acceptance as an aesthetic and patient-friendly alternative to fixed orthodontic appliances. By harnessing the elastic deformation of clear aligners (CAs) to deliver gentle, continuous forces, CAT guides teeth toward predetermined positions while offering advantages such as improved aesthetics, enhanced comfort, easier oral hygiene maintenance, and greater treatment flexibility.^1^, ^2^, ^3^

However, current clinical applications of CAT primarily address malocclusions associated with sagittal discrepancies in the dentofacial complex. For conditions involving vertical skeletal disharmony – such as high-angle malocclusions that require mandibular counterclockwise rotation – effective methods for controlling vertical intrusion, along with corresponding biomechanical evidence, remain limited. Achieving full-arch intrusion remains a significant challenge in CAT.

Traditional approaches, such as headgear traction combined with fixed appliances, have limited efficacy for mandibular repositioning. In contrast, the full-coverage design of CAs can produce an occlusal ‘bite-block effect’ on molars, utilizing the combined influence of aligner thickness and occlusal forces to inhibit or intrude posterior teeth.^4^^,^^5^ Retrospective studies have confirmed the superiority of CAs in molar vertical correction.^6^ Nevertheless, CAT often yields suboptimal outcomes in complex malocclusions, such as Class II high-angle and deep-overbite cases, which require maxillary full-arch intrusion and anterior retraction. Limitations include low vertical intrusion efficiency, inadequate anterior root torque control, and insufficient anchorage.^7^^,^^8^

The integration of traction attachments with CAs offers a promising approach to treating full-arch intrusion and complex malocclusions. As auxiliary devices, traction attachments effectively direct orthodontic forces to guide tooth movement.^9^ Digital technology studies – including medical imaging, digital model analysis, and finite element simulation – have confirmed that traction attachments significantly enhance CAT efficacy.^10^ Papadimitriou et al^11^ noted their beneficial role in tooth intrusion, while Liu and Hu^12^ demonstrated their necessity and effectiveness in premolar intrusion. Simon et al^13^ further showed that CAs combined with attachments, power ridges, and other auxiliaries can establish a favourable force system for root torque and controlled tooth movement, although perfect root control remains difficult due to anchorage limitations. Without stable anchorage, anterior teeth tend to incline palatally or exhibit root deviation during retraction, falling short of the precise biomechanical control required in complex cases.^13^

Garino et al^14^ emphasized the importance of posterior anchorage for optimal root control during anterior retraction. In a retrospective study, Wang et al^15^ compared three groups: mini-implants, Class II elastics (tooth-dependent anchorage), and no extra anchorage. They found that any extra anchorage was better than none, and that mini-implants provided superior anchorage reinforcement compared to Class II elastics. Mini-implants, once placed, provide absolute skeletal anchorage, enabling the application of targeted forces and moments without burdening adjacent teeth. This overcomes the limitations of tooth-dependent anchorage and represents an ideal anchorage modality.^16^ Celenza^17^ confirmed that mini-implants complement the biomechanical deficiencies of CAs, and subsequent studies^18^^,^^19^ support their use as effective anchorage in Class II CAT treatment.

To overcome the clinical challenge of full-arch intrusion, a combined anterior-posterior anchorage strategy using mini-implants can be adopted to establish stable anchorage in both regions. This approach creates an ‘actively controllable force system’ through a ‘multi-fulcrum adjustable force transmission’ mode, compensating for CAT’s limitations in constructing complex force systems and enhancing the predictability of vertical control. Such combined anchorage strategies have been examined in orthodontic systems other than CAs. Using finite element analysis (FEA), Korun and Topçuoğlu^20^ evaluated a configuration that combined buccal and palatal mini-implant anchorage with a palatal plate and occlusal wires uniting the posterior teeth, and found that it provided superior three-dimensional control for maxillary molar intrusion. In conventional fixed orthodontic appliances, Mazhari et al^21^ concluded that using palatal mini-implants in a combined anchorage system facilitates more effective full-arch intrusion. In contrast, within CAT, Pei and Bai^22^ reported a case in which this approach achieved successful full-arch intrusion in a patient with a gummy smile, suggesting clinical feasibility. However, to our knowledge, no additional case has been reported. Moreover, no biomechanical study has addressed such combined anchorage strategies for full-arch intrusion in CAT.

Given considerable individual variation in tooth position, difficulties in simulating periodontal environments in vitro, and the complex, nonlinear force systems involved in CAT mechanics, direct measurement via traditional experiments is challenging.^23^ FEA offers a viable alternative for calculating mechanical behaviour, such as tooth displacement and periodontal ligament (PDL) hydrostatic pressure distribution, under applied loads.^24^ FEA provides a precise, noninvasive research tool increasingly adopted in CAT biomechanical studies.^25^ Previous FEA studies have demonstrated their utility in simulating aligner mechanics: Liu et al^26^ reported a difference of less than 0.2 N between measured and FEA-calculated forces, and Ye et al^27^ demonstrated a strong correlation (*R*^2^ = 0.92) between FEA-predicted surface strain and micro-CT results. Furthermore, Penedo et al,^28^ conducting FEA of orthodontic tooth movement, found that hydrostatic pressure exceeding 2.6 kPa initiates bone remodelling and produces effective tooth movement; and Roscoe et al^29^ reported that hydrostatic pressure in the 4.7 to 12.8 kPa range further accelerates bone remodelling. Thus, FEA is an effective method for analysing orthodontic forces in CAT and for simulating the biomechanical responses of the periodontium during tooth intrusion.^30^

However, the biomechanical effects of different mini-implant anchorage configurations for maxillary full-arch intrusion with CAs have not been systematically compared. Therefore, this study aims to employ FEA to establish a combined anterior-posterior anchorage system, evaluate the influence of mini-implant placement sites on maxillary full-arch vertical intrusion, analyse associated tooth displacement and PDL hydrostatic pressure distribution, and propose an optimized anchorage protocol to inform clinical practice.

## Materials and methods

**Fig. 1:**
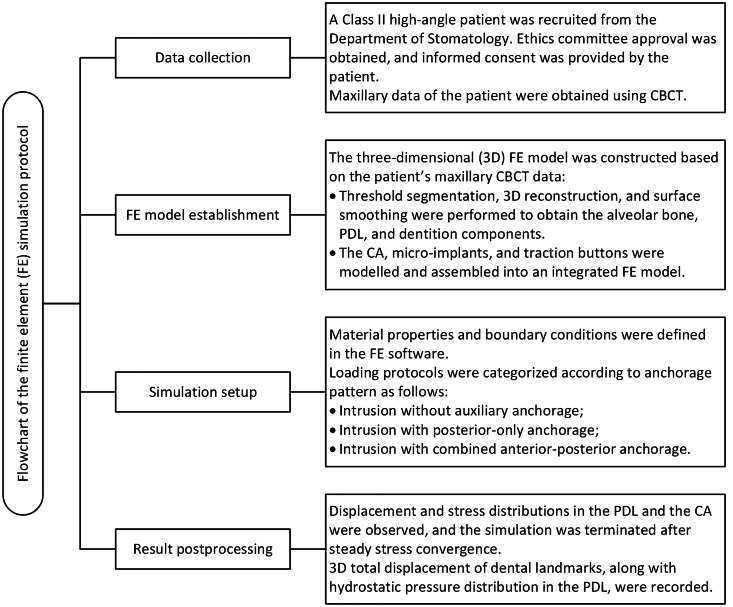
Flowchart of the study protocol.

The study workflow comprised four main stages: data acquisition, finite element model construction, simulation design, and postprocessing. A schematic overview of the workflow is presented in Figure 1.

### Subject data

**Fig. 2:**
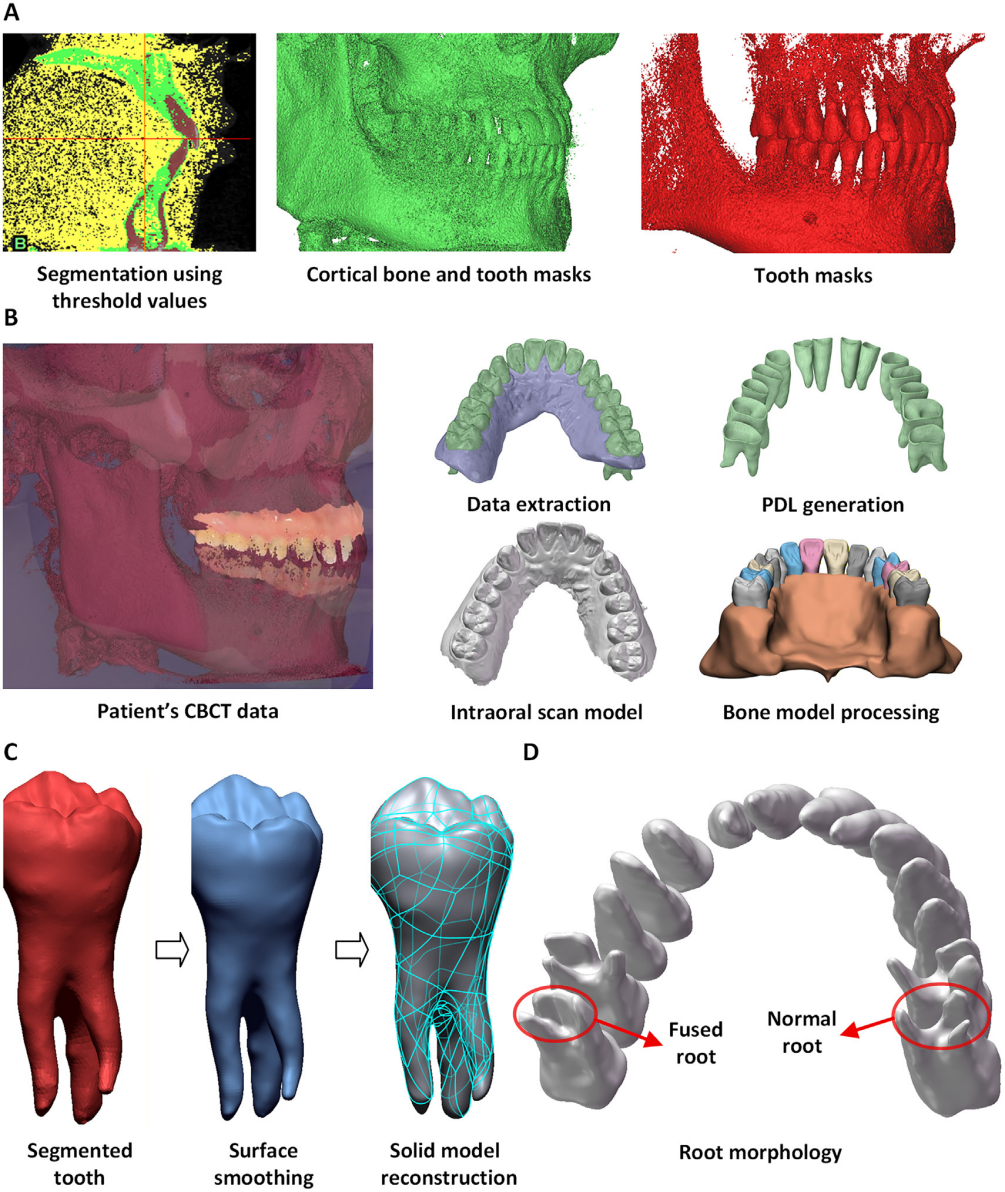
Overview of maxillary model reconstruction and root morphology: (A) data acquisition and segmentation; (B) model generation from imaging data; (C) model refinement and solid reconstruction; (D) comparison of fused and normal root morphology.

An adult patient diagnosed with Angle Class II high-angle malocclusion was recruited for this study. The patient met the following characteristics: age 22 years; no history of systemic disease or related medication use within the past 6 months; good periodontal health (probing depth ≤3 mm, with no significant alveolar bone loss); complete permanent dentition (except for the third molars); normal buccal/palatal cortical bone thickness without obvious defects; and absence of retained deciduous teeth, extensive caries, residual crowns, or roots. The patient presented with erupted maxillary molars and mandibular posterior rotation, indicating a clinical need for maxillary full-arch intrusion. Notably, tooth #27 (the second molar) exhibits a fused-root morphology (palatal and mesial root fusion, with a thick distal root) (Figure 2D), whereas tooth #17 has a normal multirooted morphology. This contrast in root anatomy introduces morphological diversity into the model, enhancing understanding of how different root configurations influence biomechanical outcomes.

Cone-beam computed tomography (CBCT) was performed from the zygomatic arch to the maxillary occlusal plane to acquire Digital Imaging and Communications in Medicine (DICOM) data of the maxillary periodontal structures.

### Model reconstruction and assembly

The DICOM data of the maxillary periodontal structures were imported into Mimics Medical 21.0 software. Initial segmentation thresholds were set based on Hounsfield unit (HU) value differences: a range of −100 to 420 HU was applied to exclude irrelevant soft tissues, while the range of 420 to 2554 HU was used to capture the maxillary cortical bone and complete dentition. Subsequently, the dentition was further isolated using a threshold of 1320 to 2554 HU to obtain a preliminary segmentation mask (Figure 2A).

Due to overlapping HU values between dental roots and cortical bone, this preliminary mask was manually refined on two-dimensional tomographic images using the PDL space as an anatomical landmark, achieving precise separation of the two structures. The segmented maxillary cortical bone and dentition were exported in stereolithography format and imported into Geomagic Design X 2020 for reverse-engineering processing, including filling surface gaps, smoothing surfaces, and generating solid models (Figure 2C). These steps yielded separate digital solid models of the maxillary cortical bone and the dentition (Figure 2B).

Building on the separate models, the cortical bone thickness was increased relative to the native CBCT-derived geometry to establish a mechanically viable shell for FEA. Following established methodology, uniformly thick layers representing the cortical bone (2 mm) and the PDL (0.25 mm) were then created using Boolean operations in Geomagic Design X 2020. Concurrently, the internal volume was defined to simulate the cancellous bone. To simplify the interfacial contact conditions, the cortical bone in this region was omitted – a simplification adopted from prior studies^31^^,^^32^ – allowing the cancellous bone to interface directly with the PDL. This series of operations ultimately produced an integrated PDL-alveolar bone-dentition assembly.

The outer crown surface was extracted from the dentition model along the gingival margin boundary to serve as the basis for the inner surface of the CA. Following the method outlined by Jia et al,^33^ a 0.75 mm outward offset was applied along the crown profile’s surface normal to generate the CA’s volumetric shape with uniform material thickness. Boolean (subtraction) operations were subsequently performed to eliminate geometric interferences between this preliminary aligner volume and the teeth, resulting in the final interference-free solid model of the aligner.

The mini-implant was modelled as a threaded stainless-steel shank with a 1.4 mm diameter and an 8 mm overall length, incorporating a supragingival cap for orthodontic traction. The traction button was designed with a total height of 2.5 mm, with a maximum base diameter of 3.5 mm and a minimum neck diameter of 1 mm. Two-dimensional layout drawings of both components were drafted in AutoCAD, followed by the development of their corresponding three-dimensional solid models in SolidWorks. All models were exported in STEP format.

**Fig. 3:**
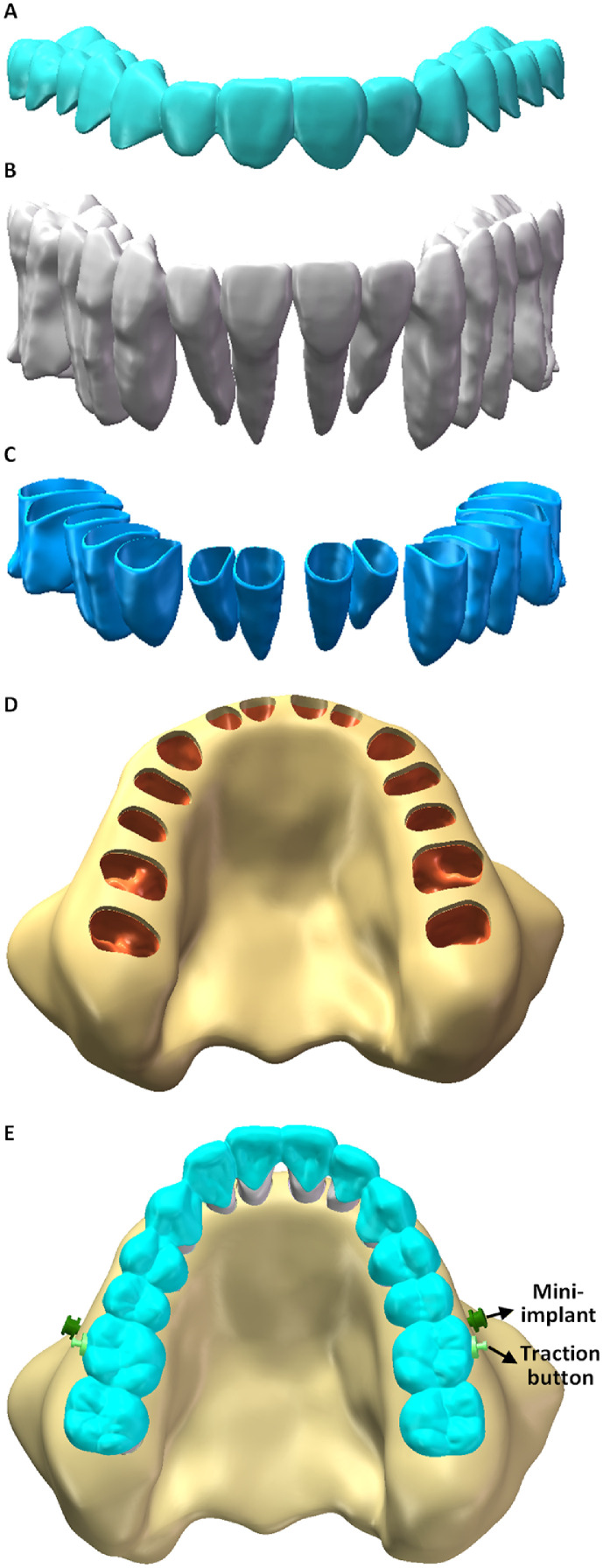
Three-dimensional finite element model of the maxillary dentition and associated components: (A) CA; (B) maxillary dentition; (C) PDL; (D) maxillary bone (cortical and cancellous); (E) overall assembly.

In Ansys, the CA, mini-implant, traction button, and the integrated PDL-alveolar bone-dentition assembly were positioned according to clinical guidelines to construct a three-dimensional finite element model representing the in vivo scenario (Figure 3). Contact conditions were defined at relevant tissue interfaces, and the SpaceClaim module was used to identify and resolve geometric interferences, yielding a validated digital model ready for mechanical analysis.

To reflect the clinical implant position, each mini-implant was placed 8 mm apical to the alveolar crest and oriented at a 30° angle relative to the long axis of the adjacent tooth. This site offered the greatest interradicular space and helped avoid soft tissue coverage.

Traction buttons were bonded at the midcoronal level of the teeth ipsilateral to the mini-implants. When mini-implants were placed between the second premolar and the first molar, traction buttons were attached at the midcoronal level of the first molar.

In the anterior region, the positions of the traction buttons were adjusted to align with the intended intrusive force vector, thereby optimizing the direction of tooth movement. Specifically, when a mini-implant was inserted between the central incisors, traction buttons were placed at the midcoronal level of each central incisor; when mini-implants were placed between the lateral incisors and the canines, traction buttons were positioned at the midcoronal level of each lateral incisor.

### Boundary conditions and material properties

In this study, the CA, alveolar bone (cortical and cancellous), and dental hard tissues were modelled as isotropic, homogeneous, linear-elastic materials.^34^^,^^35^ Their Young’s moduli and Poisson’s ratios are summarized in Table 1, consistent with previously reported values.^31^^,^^36^ For the PDL, in addition to the elastic modulus and Poisson’s ratio listed in Table 1, a three-term Prony series viscoelastic constitutive model was adopted to capture its stress relaxation and creep behaviour under orthodontic loading. The corresponding viscoelastic coefficients are listed in Table 2.^37^

**Table 1:** Material properties and mesh characteristics of the components in the finite element model.

Components	Young’s modulus (MPa)	Poisson’s ratio	Element count	Node count
Teeth	20,000	0.30	126,762	224,886
PDL	0.71	0.42	77,359	156,833
Cortical bone	13,700	0.30	25,202	51,217
Cancellous bone	1370	0.30	125,430	211,468
Microimplants	206,000	0.32	17,949	70,151
Traction buttons	2000	0.34	17,468	68,170
CA	816	0.36	116,117	207,757

**Table 2:** Three-term Prony series coefficients for the PDL.

Term	Relative modulus	Relaxation time (s)
1	0.91	0.0025
2	0.05	0.1
3	0.005	0.5

To realistically simulate the compliance of the maxillary base and avoid artificial stress concentration in the PDL, the bone support was modelled using a Winkler elastic foundation approach.^38^ According to the Winkler foundation model with two layers in series, the compliances of the cortical and cancellous bone layers are superimposed in series. The equivalent stiffness *k*_eff_ of the thin alveolar bone region is calculated from the thickness (*h*_cort_, *h*_canc_) and elastic modulus (*E*_cort_, *E*_canc_) of each layer:

(1) $$k_{\text{eff}}={\left(\frac{h_{\text{cort}}}{E_{\text{cort}}}+\frac{h_{\text{canc}}}{E_{\text{canc}}}\right)}^{-1}$$

Using representative values for cortical and cancellous bone, this yields a theoretical value of approximately 428 N/mm^3^. Considering the actual variation in bone thickness, a final foundation stiffness of 500 N/mm^3^ was adopted.

The contact between the CA and the teeth was defined as frictionless surface-to-surface contact. Bonded contacts were assigned to all interfaces between the cancellous bone, cortical bone, PDL, teeth, and mini-implants, as well as between the traction buttons and the CA.

Mesh generation was conducted in HyperMesh 14.0. Tetrahedral elements were used for the CA, teeth, and alveolar bone due to their anatomical complexity, whereas hexahedral elements were applied to the traction buttons and mini-implants owing to their relatively simple, regular geometry. Mesh quality was assessed and confirmed, with an average aspect ratio of 1.16 and an element quality of 0.86 – both values close to 1, indicating good mesh quality. The final mesh consisted of 990,482 nodes and 506,287 elements; detailed node and element counts for each component are provided in Table 1.

### Study groups and loading scheme

**Fig. 4:**
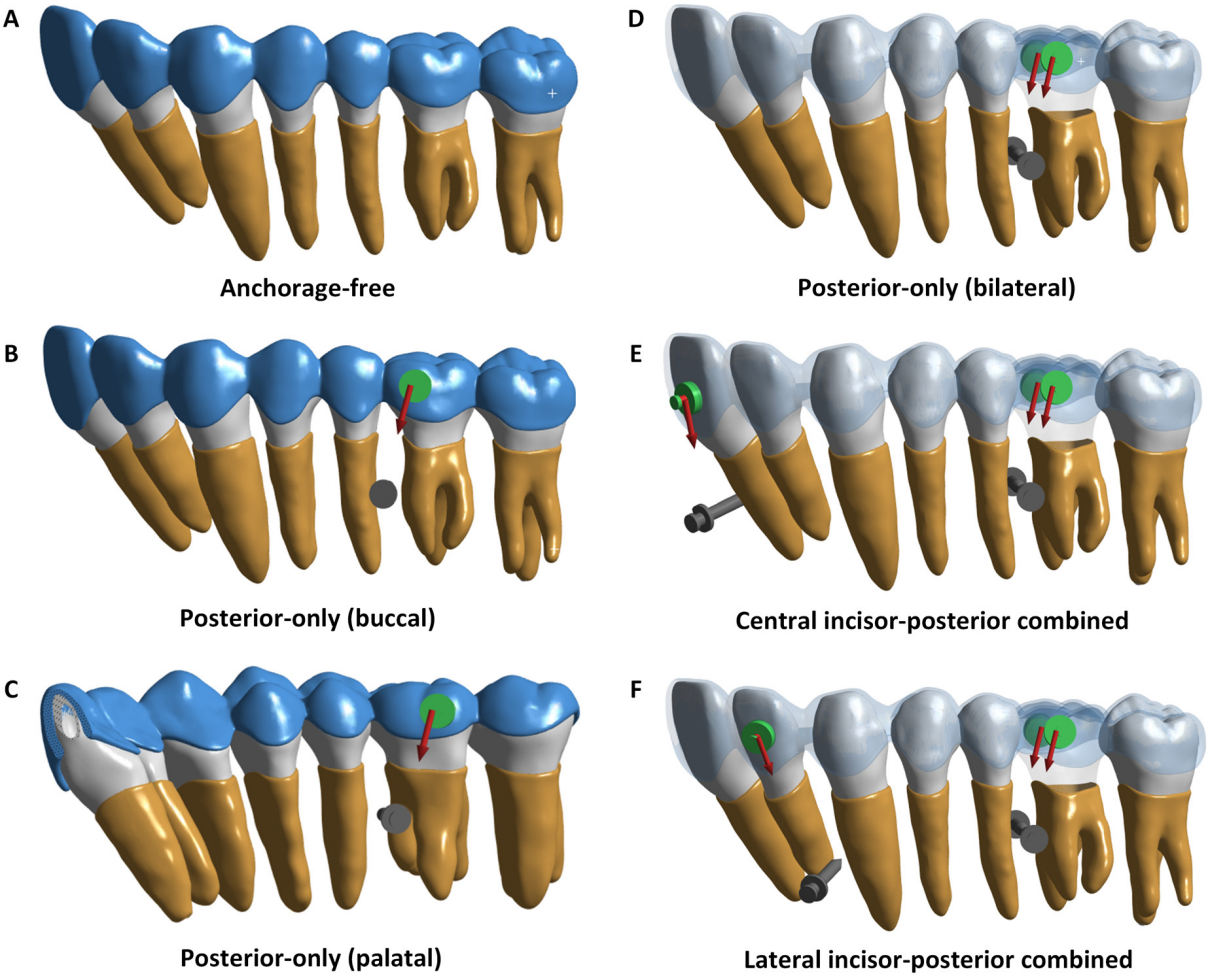
Anchorage configurations of (A) control group, (B-D) posterior-only anchorage groups, and (E and F) combined anterior-posterior anchorage groups: (B) buccal-only; (C) palatal-only, and (D) bilateral; (E) central incisor-posterior; (F) lateral incisor-posterior.

This study employed FEA to evaluate the biomechanical efficacy of mini-implant-assisted intrusion of the maxillary dentition, targeting a clinical intrusion of 0.2 mm. The simulations were organized into three anchorage categories: (1) intrusion without auxiliary anchorage (Figure 4A), (2) intrusion with posterior-only anchorage (Figure 4B-D), and (3) intrusion with combined anterior-posterior anchorage (Figure 4E and F).

A systematic review on molar intrusion by Manea et al^39^ found that most studies recommend a traction force of 50 to 200 gf for mini-implant anchorage, with a single case reporting a higher force of 300 gf as an exception. In the present study, categories 2 and 3 utilized a force of 100 gf per posterior mini-implant in unilateral configurations, and 50 gf per posterior mini-implant in bilateral configurations. For the anterior anchorage in category 3, a force of 50 gf per traction chain was applied to the mini-implant. All selected forces are within the clinically recommended range reported by Manea et al.^39^ Anchorage configurations and loading schemes for these tested groups are summarized in Table 3, while the force mechanism for the anchorage-free group (category 1) is detailed in the section ‘Intrusion without auxiliary anchorage’.

**Table 3:** Anchorage configurations and loading schemes.

Group	Anchorage-free	Posterior-only (buccal)	Posterior-only (palatal)	Posterior-only (bilateral)	Central incisor-posterior combined	Lateral incisor-posterior combined
Anchorage position	-	#5/6	#5/6	#5/6	#11/21, #5/6	#2/3, #5/6
Number of mini-implants	-	2	2	4	5	6
Number of traction chains	-	2	2	4	6	6
Force per traction chain	-	100 gf (0.98 N)	100 gf (0.98 N)	50 gf (0.49 N)	50 gf (0.49 N)	50 gf (0.49 N)
Total traction force	-	200 gf (1.96 N)	200 gf (1.96 N)	200 gf (1.96 N)	300 gf (2.94 N)	300 gf (2.94 N)

#### Intrusion without auxiliary anchorage

CAT without auxiliary anchorage cannot achieve simultaneous intrusion of all teeth in the arch. Although no clinical application has been reported, a theoretical stepwise, tooth-by-tooth intrusion protocol could be envisaged. Owing to their multirooted morphology and complex biomechanical behaviour, molars present the greatest challenge for intrusion. Accordingly, this study evaluated the molar intrusion capacity of anchorage-free aligners as a control group against which mini-implant-assisted approaches were compared.

**Fig. 5:**
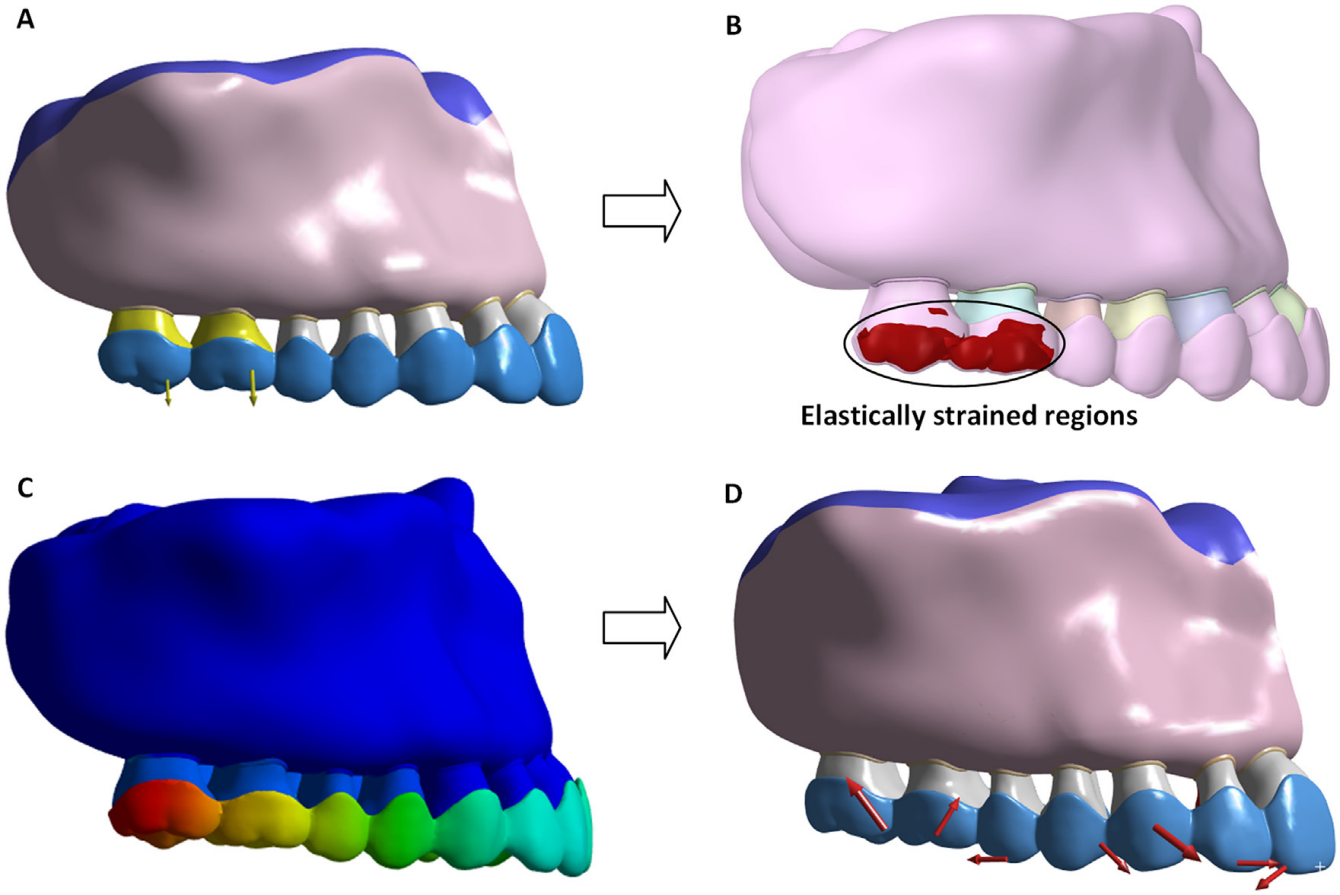
Force application protocol for the control group: (A) initial extrusive displacement of teeth #6 and #7 by 0.2 mm with the CA held in place; (B) elastic deformation of the CA induced by tooth displacement; (C) measurement of elastic rebound forces at each tooth-aligner interface via postprocessing; (D) reverse application of the measured forces to the corresponding contact surfaces.

The force-loading method followed the approach of Jiang et al.^32^ The maxillary first and second molars (#6 and #7) were initially displaced upward by 0.2 mm while the CA remained in place (Figure 5A). This displacement induced elastic deformation of the seated aligner (Figure 5B). Once static equilibrium between the deformed aligner and the teeth was reached, the resultant elastic rebound force was measured using a force probe in the ANSYS postprocessing module. This force was then applied in the reverse direction to the corresponding tooth-aligner contact interfaces (Figure 5C). The gap created by the upward displacement of the teeth was filled with material representing cancellous bone, thereby simulating isolated posterior intrusion by the aligner alone (Figure 5D).

#### Intrusion with posterior-only anchorage

For posterior-only anchorage, the force system was designed so that its line of action passed near the dentition’s centre of resistance to facilitate bodily vertical intrusion. Finite-element studies indicate that the centre of resistance of the maxillary dentition lies near the midcoronal level of the second premolar.^40^ Therefore, mini-implants were placed between the second premolar and first molar (#5/#6) as posterior anchorage. As shown in Figure 4B-D, three groups were defined: (A) buccal-only group (two buccal implants), (B) palatal-only group (two palatal implants), and (C) bilateral group (four implants, two buccal and two palatal). In all three groups, elastic chains connected the mini-implants to traction buttons to deliver the orthodontic force.

#### Intrusion with combined anterior-posterior anchorage

Combined anchorage was implemented by adding anterior anchorage to the bilateral posterior setup, introducing an additional intrusive component to the anterior teeth. This design aimed to distribute intrusive force more uniformly across the entire arch, thereby reducing compensatory displacements and adverse reactions caused by uneven force distribution.

The design of the combined anchorage configurations was informed by prior finite-element and clinical studies. In the context of anterior tooth retraction using fixed appliances, Namburi et al^41^ adopted central incisor anchorage combined with molar anchorage and concluded that central incisor anchorage enhances the retraction and intrusion of anterior teeth. For full-arch intrusion with fixed appliances, Mazhari et al^21^ used lateral incisor plus molar anchorage, focusing on moment balance between anterior and posterior teeth. In CAT, a recent case study by Pei and Bai^22^ addressed maxillary gummy smiles using a staged mini-implant placement protocol: four mini-implants were inserted in the maxillary molar region at the third aligner stage (out of 47 aligners), and mini-implants were subsequently placed labially between the roots of the incisors and canines at the 15th aligner stage to facilitate anterior intrusion.

Drawing on these findings, two configurations were tested in this study (Figure 4E and F): (A) central incisor-posterior anchorage, with one mini-implant placed labially between the central incisors; and (B) lateral incisor-posterior anchorage, with one mini-implant placed labially between the lateral incisor and canine on each side (#12/#13 and #22/#23).

Orthodontic forces were applied via elastic chains attached to the traction buttons. Due to the limited bone thickness on the palatal side in the anterior region, mini-implants were placed only on the labial side.

### Finite element simulation and data analysis

Mechanical responses under the prescribed orthodontic loads were simulated in ANSYS, incorporating the boundary conditions and constraints defined in previous sections. Each simulation was run until global force equilibrium and numerical convergence were achieved. The analysis assessed the three-dimensional displacement of the maxillary teeth and the hydrostatic pressure in the PDL.

## Results

### Tooth displacement with posterior-only anchorage

**Fig. 6:**
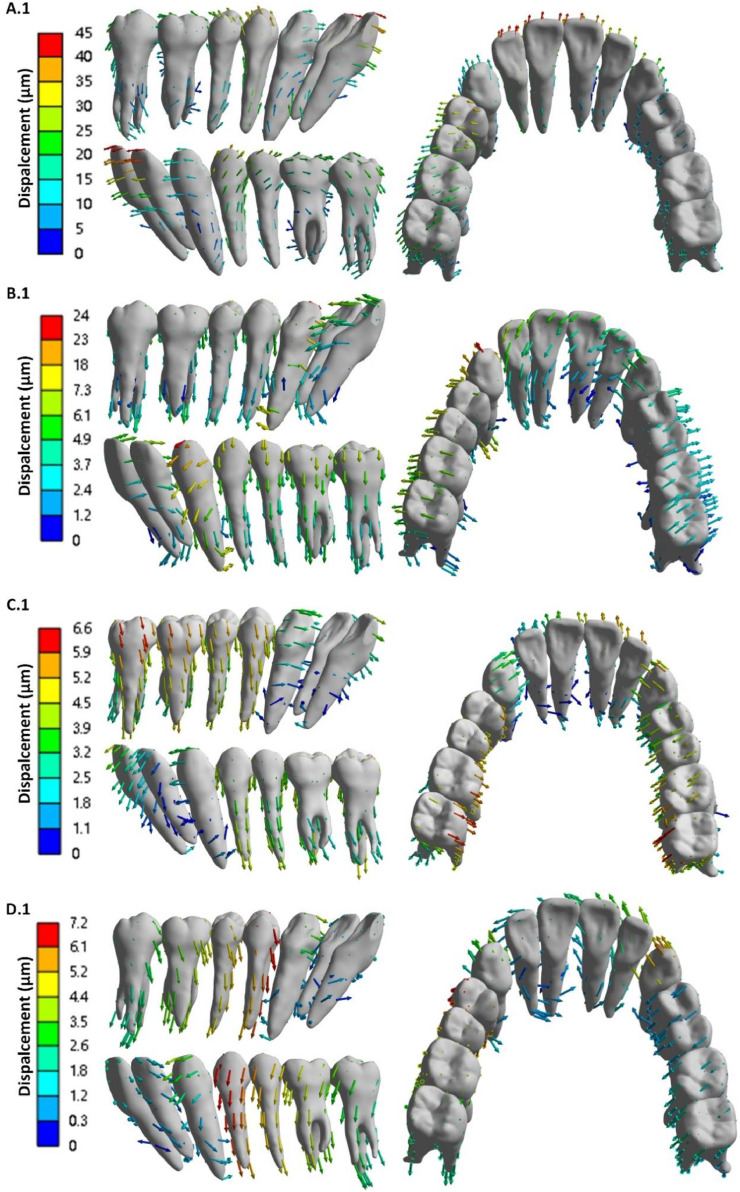

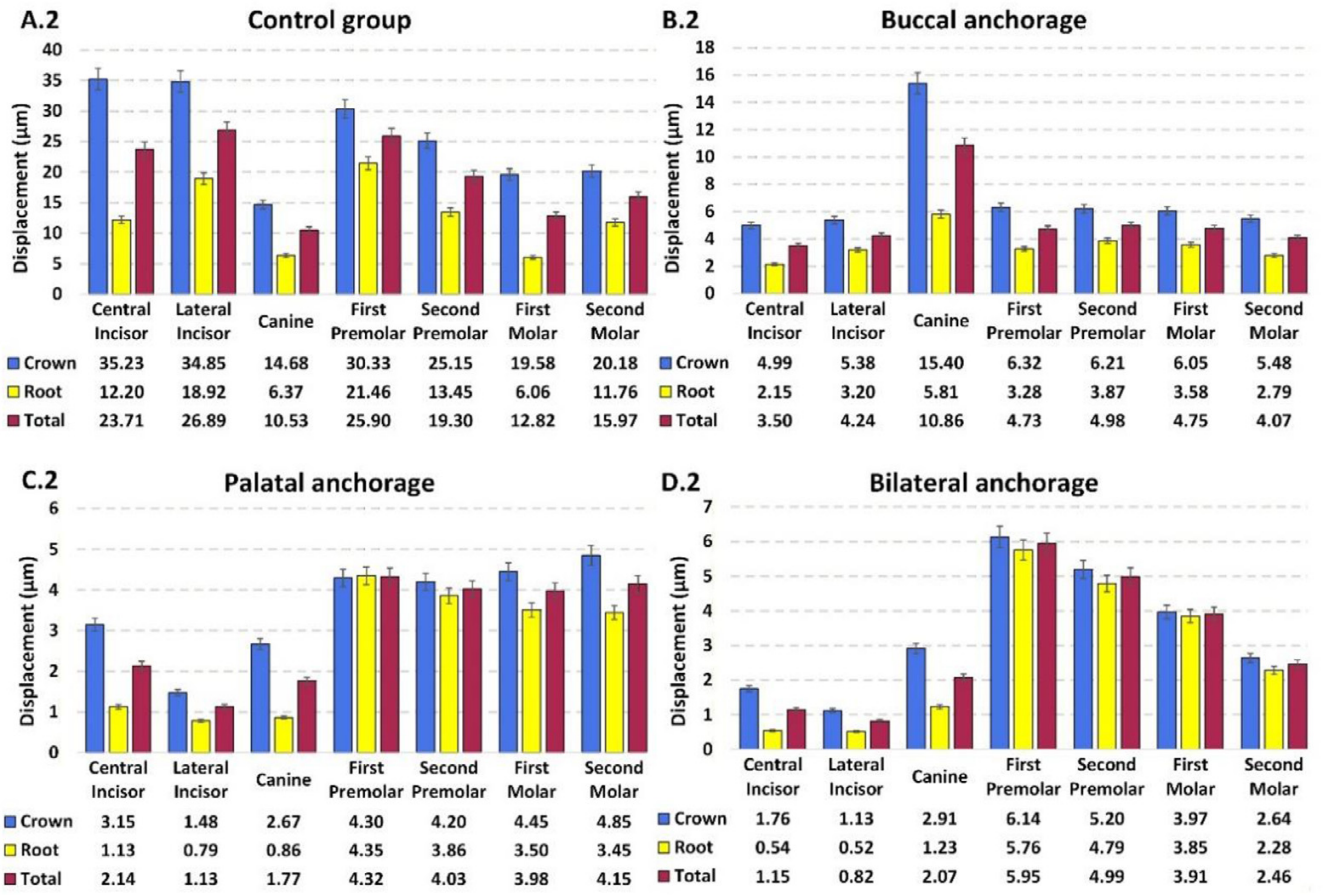
Tooth displacement patterns under anchorage-free and different posterior-only anchorage configurations (mini-implants placed between teeth #5 and #6): (A) control group; (B) buccal-only anchorage; (C) palatal-only anchorage; (D) bilateral anchorage. Subfigures (A.1, B.1, C.1, and D.1) show the displacement trend of teeth; subfigures (A.2, B.2, C.2, and D.2) present the mean values of crown displacement, root displacement, and total displacement calculated from 20 evenly distributed sampling points per tooth. All displacement results shown in this figure were obtained from the normally rooted first-quadrant teeth.

Displacement analysis was performed by sampling 20 points per tooth (10 on the crown and 10 on the root). As shown in Figure 6, for each anchorage configuration, subfigures A.1, B.1, C.1, and D.1 present the displacement trend as coloured vectors, while subfigures A.2, B.2, C.2, and D.2 show the mean values of crown, root, and total displacement.

In the control group (Figure 6A), the anterior teeth exhibited labial tipping, while the posterior teeth showed a combination of intrusive displacement and distal rotation, with marked asynchrony between crown and root movement – indicating suboptimal root control with this nonanchored approach.

In the buccal-only anchorage group (Figure 6B), the posterior teeth exhibited intrusive displacement accompanied by buccal crown tipping, while the anterior incisors showed palatal inclination, and the canines demonstrated labial inclination. Compared with the anterior teeth (excluding the canines), the posterior teeth showed greater displacement, with the buccopalatal displacement directed buccally. Across the entire dental arch, crown displacement exceeded root displacement, indicating that tipping movement was predominant. Among all teeth, the canines exhibited the greatest displacement values.

In the palatal-only anchorage group (Figure 6C), the posterior teeth exhibited intrusive displacement accompanied by palatal crown tipping, while the anterior teeth showed labial inclination. Across the posterior teeth, the buccopalatal displacement was palatally directed. Compared with the buccal-only anchorage group, the intrusive displacement of the posterior teeth was more evenly distributed, and the sagittal inclination of the anterior teeth was reduced.

In the bilateral anchorage group (Figure 6D), posterior teeth showed bodily displacement, as evidenced by nearly equal movement of the crown and root, indicating predominantly intrusive movement with minimal crown tipping.

Among the four groups, the bilateral anchorage group showed the most bodily intrusion of posterior teeth and the smallest sagittal inclination of anterior teeth, demonstrating the most favourable root control. However, across all three posterior anchorage-assisted groups, a consistent finding in the anterior region was that canine movement behaviour remained relatively difficult to control.

### Tooth displacement with combined anterior-posterior anchorage

**Fig. 7:**
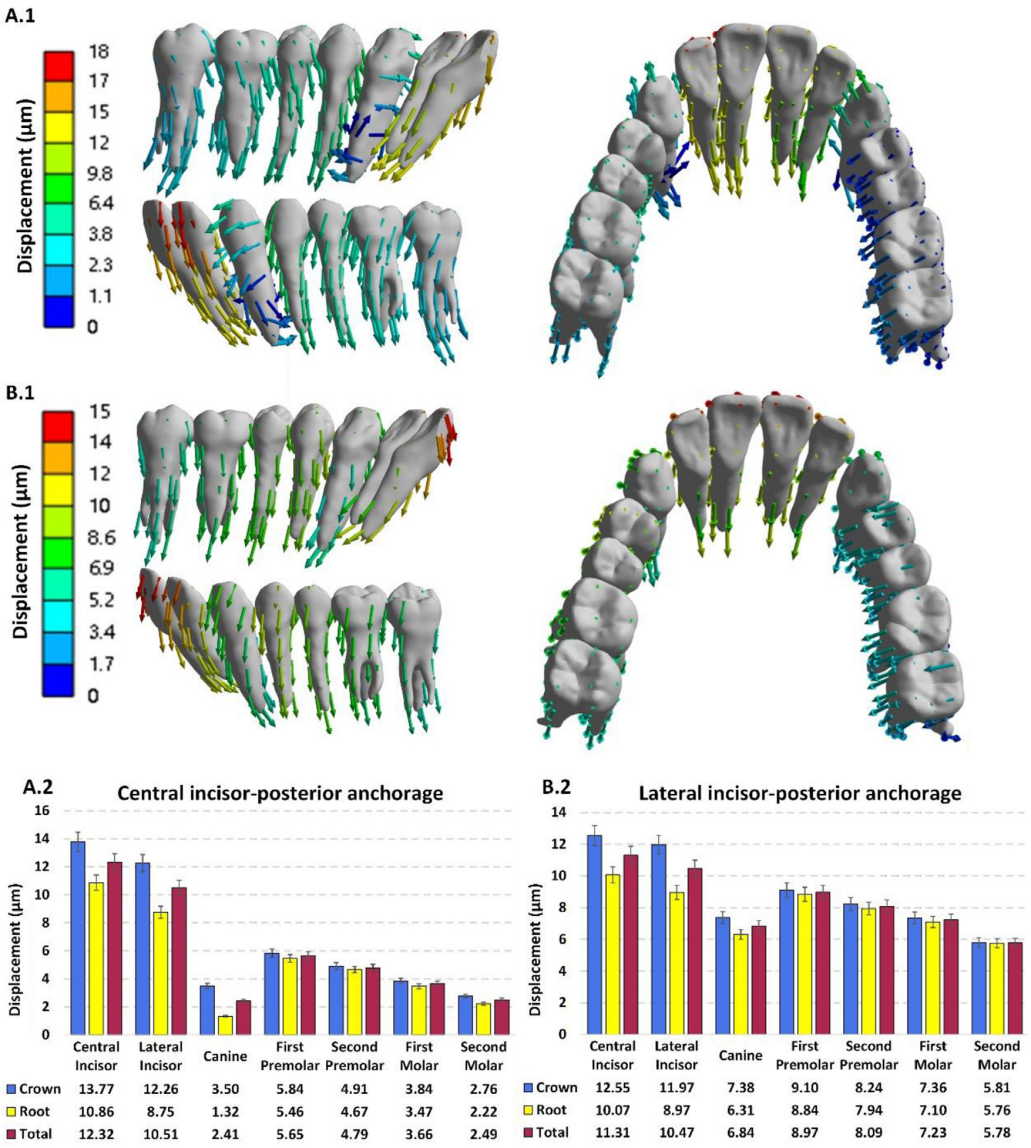
Tooth displacement patterns under two combined anterior-posterior anchorage configurations: (A) central incisor-posterior anchorage; (B) lateral incisor-posterior anchorage. Subfigures (A.1 and B.1) show the displacement trend of teeth; subfigures (A.2 and B.2) present the mean values of crown displacement, root displacement, and total displacement calculated from 20 evenly distributed sampling points per tooth. All displacement results shown in this figure were obtained from the normally rooted first-quadrant teeth.

Combined anterior-posterior anchorage was implemented by adding anterior mini-implants to the bilateral posterior setup. Two configurations were tested (Figure 7): central incisor-posterior anchorage, with a mini-implant placed labially between the central incisors (#11/#21) (Figure 7A), and lateral incisor-posterior anchorage, with mini-implants placed labially between the lateral incisor and canine on each side (#12/#13 and #22/#23) (Figure 7B).

In the central incisor-posterior anchorage group (Figure 7A), the total displacement of the posterior teeth increased compared with bilateral posterior anchorage alone, and vertical displacement became the dominant component of overall tooth movement. The primary displacement pattern of the anterior teeth (excluding the canines) shifted from rotation to intrusion aligned with the long axis of the teeth, with greater intrusion magnitude. The maximum displacement consistently occurred at the incisal crowns. However, this configuration provided only limited vertical control over the canines, and overall arch intrusion tended to be deeper in the anterior region.

In the lateral incisor-posterior anchorage group (Figure 7B), effective full-arch intrusion was achieved. Under the same magnitude of external force, this configuration exhibited greater displacement than the central-incisor group, and the intrusion was more bodily for each tooth across the arch.

These findings indicate that adding anterior anchorage to a bilateral posterior setup enhances vertical control of the anterior dentition, with the lateral incisor-posterior anchorage configuration yielding more favourable full-arch intrusion than the central incisor-posterior anchorage configuration.

### Hydrostatic pressure in PDL

The PDL is a dense connective tissue that anchors the tooth root to the alveolar bone. Under orthodontic loading, mechanical stimulation induces biological responses, including alveolar bone resorption and apposition, thereby facilitating tooth movement. Excessive hydrostatic pressure within the PDL may also contribute to root resorption.

In this study, hydrostatic pressure within the PDL was used to evaluate the magnitude of mechanical stimulation and to reflect the intensity of bone remodelling. Positive hydrostatic pressure was defined as hydrostatic compression, which may promote osteoclastic activity, bone resorption, and tooth intrusion, whereas negative values represented hydrostatic tension, which may promote bone formation and tooth extrusion. In the following analysis, the term hydrostatic pressure refers specifically to positive hydrostatic pressure (hydrostatic compression).

**Fig. 8:**
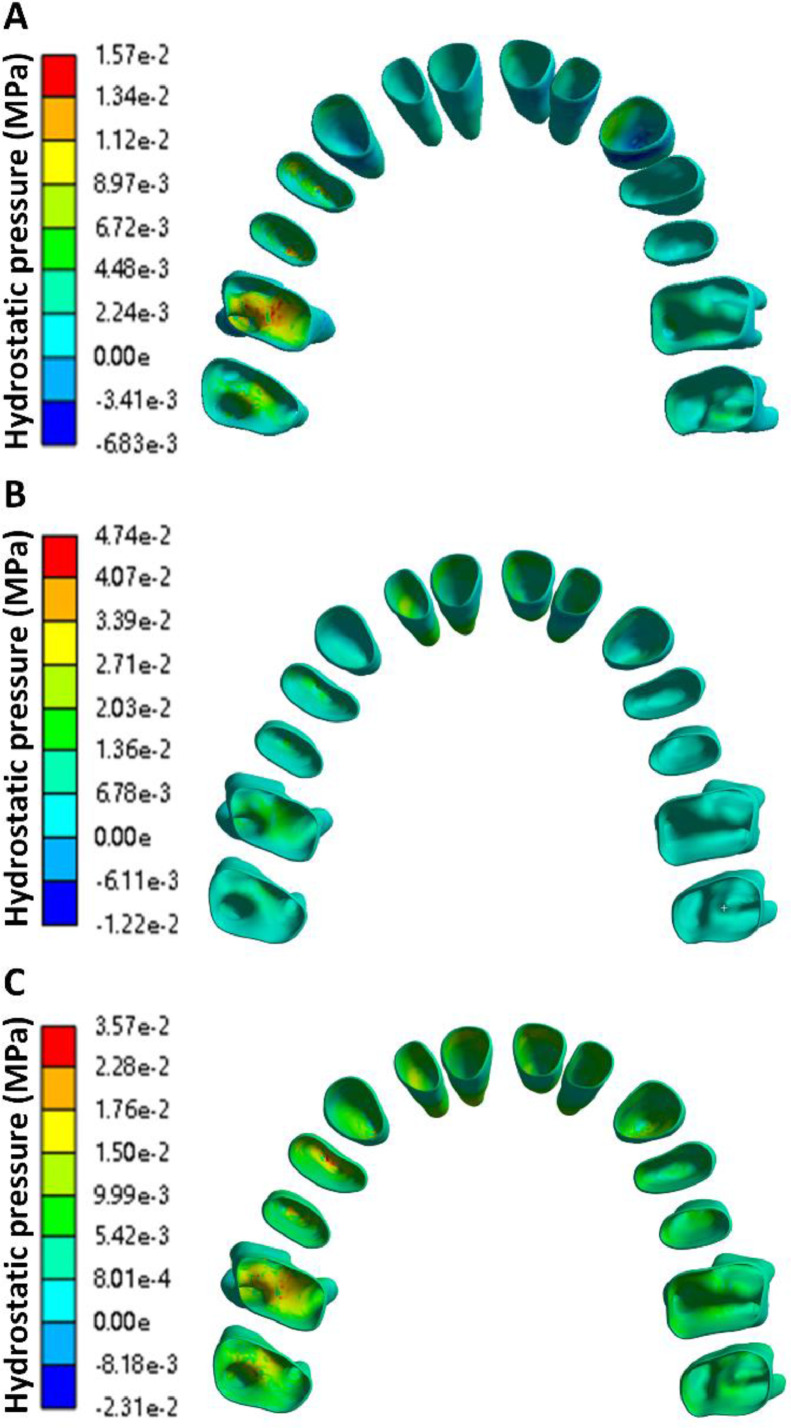
Distribution of hydrostatic pressure in the PDL across all 14 maxillary teeth under the three bilateral-posterior-involved anchorage configurations: (A) bilateral posterior anchorage alone; (B) central incisor-posterior anchorage; (C) lateral incisor-posterior anchorage.

Hydrostatic pressure values for each tooth were visualized as nephograms generated by the ANSYS postprocessing module. Figure 8 shows the distribution of hydrostatic pressure in the PDL across all 14 maxillary teeth under the three anchorage configurations. Based on these results, subsequent trends in biological bone remodelling could be predicted.

In the bilateral posterior anchorage group (Figure 8A), hydrostatic pressure was predominantly distributed at the apical region of the central incisors and posterior teeth, and at the labial cervical region of the lateral incisors and canines. The peak hydrostatic pressure occurred at the first-molar apex, with a value of 1.56 × 10^−2^ MPa. The mean hydrostatic pressure was 2.64 × 10^−3^ MPa in the apical region of posterior teeth, 1.33 × 10^−3^ MPa in the central-incisor apical region, and 1.58 × 10^−4^ MPa in the labial cervical region of the lateral incisors and canines. The hydrostatic pressure results suggest that bone resorption is likely in the posterior apical region, whereas pressure in the anterior teeth remains below the threshold required to trigger bone remodelling. This predicted bone remodelling indicates that the posterior teeth primarily undergo intrusion, with the anterior teeth experiencing only minimal displacement.

In the central incisor-posterior anchorage group (Figure 8B), PDL hydrostatic pressure in the anterior region increased markedly. Hydrostatic pressure was predominantly distributed at the apex and labial cervical third of the anterior teeth, and at the root furcation and apex of the posterior teeth. The peak hydrostatic pressure occurred at the labial apex of the central incisors, with a value of 4.03 × 10^−2^ MPa. The arch-wide mean hydrostatic pressure in the apical region was 3.35 × 10^−3^ MPa. These results indicate that bone resorption is expected at the apex and labial cervical region of anterior teeth, as well as at the root furcation and apex of posterior teeth. This predicted bone remodelling shows intrusion in the posterior teeth, and intrusion accompanied by labial tipping in the anterior teeth.

In the lateral incisor-posterior anchorage group (Figure 8C), hydrostatic pressure in every tooth was predominantly distributed at the apical region. The peak hydrostatic pressure occurred at the first-premolar apex, with a value of 3.46 × 10^−2^ MPa. The arch-wide mean hydrostatic pressure in the apical region was 5.38 × 10^−3^ MPa. These results indicate that bone resorption is expected at the apical region of every tooth, predicting full-arch intrusion.

### Displacement and hydrostatic pressure differences between quadrants with different molar root morphologies

**Fig. 9:**
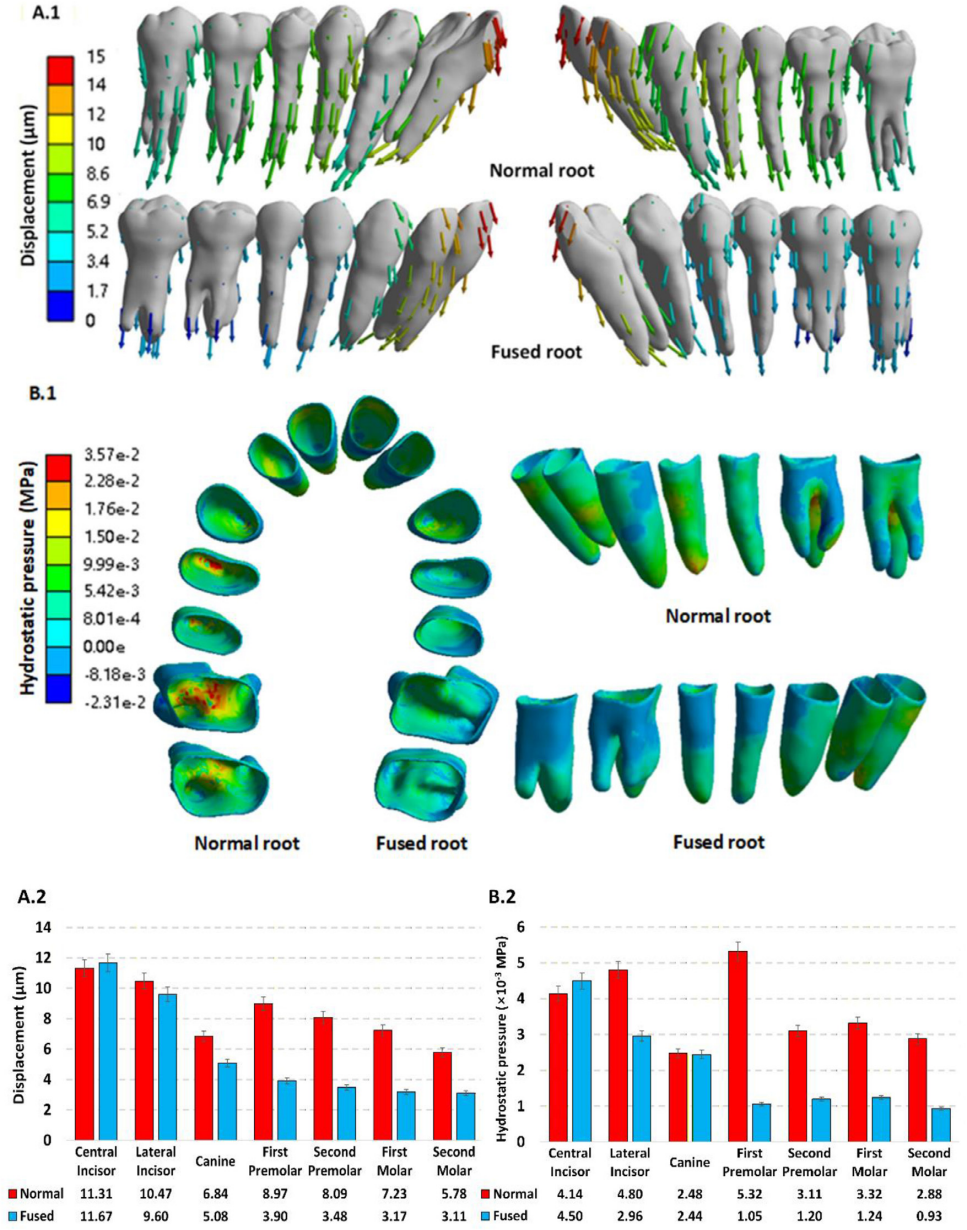
Comparison of displacement and hydrostatic pressure distribution between first-quadrant teeth (with normally-rooted second molar) and second-quadrant teeth (with fused-root second molar) under lateral incisor-posterior anchorage: (A) tooth displacement; (B) PDL hydrostatic pressure.

To investigate the influence of a fused-root second molar on the biomechanical response of the entire quadrant, a comparison was made of displacement (Figure 9A) and hydrostatic pressure (Figure 9B) between the first quadrant (with a normally-rooted second molar) and the second quadrant (with a fused-root second molar) under lateral incisor-posterior anchorage. Aside from the second molar, the remaining teeth showed no major anatomical differences between the two quadrants.

Under identical loading, anterior displacement showed little difference between the two quadrants, whereas posterior displacement was substantially lower in the second quadrant (fused-root group). Among the four posterior teeth, the first premolar exhibited the greatest reduction in intrusive displacement, decreasing from 8.97 μm in the first quadrant to 3.90 μm in the second quadrant – a decrease of 56.5%.

Regarding hydrostatic pressure, compared with the first quadrant, the mean PDL hydrostatic pressure in the second quadrant remained similar in the central incisors and canines but decreased in the remaining teeth, with the most pronounced reductions in the posterior teeth. The mean PDL hydrostatic pressure of the first premolar decreased from 5.32 × 10^−3^ MPa to 1.05 × 10^−3^ MPa (a reduction of 80.3%), and that of the second molar from 2.88 × 10^−3^ MPa to 0.93 × 10^−3^ MPa (a reduction of 67.7%).

The biomechanical implications of these findings will be addressed in the section ‘Asymmetric displacement and hydrostatic pressure between quadrants’.

## Discussion

### Posterior intrusion under different anchorage configurations

In the anchorage-free group (control group), the anterior teeth exhibited labial tipping, while the posterior teeth showed a combination of intrusive displacement and distal rotation. This result reflects the inherent force characteristics of CAs in the absence of auxiliary anchorage. Without anchorage support, the orthodontic force generated by elastic deformation of the aligner is passive in nature, and its line of action cannot be precisely directed through the centre of resistance of the arch or individual teeth. For posterior teeth, deviation of the line of action readily induced distal tipping. For anterior teeth, the combination of thin alveolar bone and an uneven distribution of passive intrusive force resulted in a mild sagittal inclination, failing to achieve bodily vertical intrusion. Moreover, the unpredictable force magnitude in the anchorage-free group – coupled with the inability to adjust force direction without stable anchorage – further exacerbated movement instability, triggering compensatory root deviation and further compromising root control. This finding aligns with previous studies,^7^ which have identified insufficient anchorage as the primary cause of poor tooth movement control in CAT.

When orthodontic force was applied unilaterally via mini-implants confined to the posterior region (buccal-only or palatal-only), the posterior teeth underwent vertical displacement accompanied by significant coronal tipping – specifically, the crown tilted toward the side of force application around the centre of resistance. Simultaneously, the anterior region experienced compensatory displacement secondary to sagittal anchorage loss. This finding is consistent with previous studies,^32^^,^^42^, ^43^, ^44^ which reported that uncontrolled tipping of the anterior teeth, particularly the incisors, is common. This phenomenon may be explained by the ‘bowing effect’.

Notably, when bilateral simultaneous force was applied with 50 gf on each side (buccal and palatal), tipping of the posterior teeth was significantly reduced, resulting in primarily vertical displacement of these teeth. This suggests that buccal-palatal moment balance is critical for achieving controlled, bodily intrusion of the posterior teeth. Although the wraparound structure of the CA provides some crown constraint during vertical loading, it remains insufficient to counteract the lateral moment generated by unilateral force. Clinically, therefore, bilateral anchorage implants with simultaneous force application should be preferred when uniform posterior intrusion is desired, as this approach minimizes undesirable tipping and potential occlusal interference.

In the posterior-only anchorage groups, palatal-only anchorage produced less tipping and more bodily intrusion than buccal-only anchorage.^45^ This may be explained by the anatomical location of the combined centre of resistance of the adult posterior teeth (#5 and #6), which is situated at the furcation area of the first molar. Owing to the longer palatal root and greater bone volume on the palatal side, this centre of resistance lies closer to the palatal mini-implant in the buccopalatal dimension. Consequently, the line of action of the traction force more closely approximates the centre of resistance, generating a smaller tipping moment on the posterior teeth. In contrast, under buccal anchorage, the line of action is farther from the centre of resistance, making tipping more likely during intrusion. Similar findings have been reported in studies.^31^^,^^46^

### Full-arch intrusion under combined anchorage configurations

Achieving optimal outcomes with CAT relies critically on the stability of the anchorage system.^46^ Following the addition of anterior anchorage to the bilateral posterior setup, the arch exhibited more bodily vertical displacement, and the sagittal tipping of the anterior teeth was mitigated. Notably, under the combined anchorage configuration of lateral incisors and posterior teeth, significant intrusion of the full arch was achieved. These findings are further supported by the clinical case reported by Pei and Bai,^22^ which also demonstrated successful full-arch intrusion using a similar approach.

These findings suggest that strategic placement of mini-implants in the anterior region can prevent unwanted extrusion of anterior teeth while concurrently facilitating posterior intrusion, thereby enabling coordinated vertical control. From a mechanical standpoint, the combined anterior-posterior anchorage creates a two-point fixation system that enhances mechanical stability. During posterior intrusion, the reactive forces are counteracted by the anterior anchorage, preserving the vertical position of the anterior teeth, and the reciprocal effect occurs during anterior loading. The results corroborate the hypothesis that the combined anchorage design substantially improves the controllability of orthodontic force transmission.^20^^,^^21^

It should be particularly noted that the elastic displacement calculated by FEA is orders of magnitude smaller than the clinical target for tooth movement. This does not indicate an error in the simulation; rather, it reflects that FEA captures the immediate elastic deformation and stress distribution within the periodontal tissues under orthodontic loading, whereas actual orthodontic tooth movement results from a time-dependent bone remodelling process. These two phenomena are distinct yet closely linked: the elastic deformation induced by orthodontic force generates hydrostatic pressure within the PDL. When this pressure exceeds a critical threshold, it triggers alveolar bone resorption or formation, which in turn drives tooth displacement. Therefore, although the absolute elastic displacement cannot be directly equated with clinical tooth movement, the calculated displacement patterns and PDL hydrostatic pressure provide reliable predictions of the location, direction, and extent of bone remodelling, and consequently of the direction and magnitude of orthodontic tooth movement.

### Influence of anchorage configuration on PDL hydrostatic pressure and intrusion efficiency

Moderate, evenly distributed PDL hydrostatic pressure promotes alveolar bone remodelling and stable tooth movement, whereas localized hydrostatic pressure elevation increases the risk of root resorption. When hydrostatic pressure within the PDL exceeds local capillary pressure, vascular compromise may occur, leading to periodontal necrosis and root resorption.^47^ Hydrostatic pressure is also the most reliable biomechanical indicator for predicting tooth intrusion^48^: pressures above 2.60 kPa initiate bone remodelling and produce intrusion^28^; within the 4.7 to 12.8 kPa range, tooth displacement accelerates; once hydrostatic pressure exceeds 12.8 kPa, the risk of root resorption rises markedly.^49^^,^^50^

Against this background, the present results showed that PDL hydrostatic pressure was consistently elevated at the root apex and cervical region across the intruded arch – precisely the sites where root resorption is most frequently observed clinically.^47^^,^^51^ This pattern reflects common features of root anatomy: roots taper toward the apex and narrow at the cementoenamel junction, so the PDL attachment area is smallest at these sites. Thus, under equivalent loading, these regions experience higher hydrostatic pressure than the midroot, producing pronounced peaks. The effect was most extreme in the central incisor-posterior anchorage group, where the slender, conical incisor root and narrow cervical zone drove the peak hydrostatic pressure at the central-incisor apex to ≈40.3 kPa. In the lateral incisor-posterior anchorage group, the peak reached ≈34.6 kPa at the first-premolar apex. Both values lie well above the 12.8 kPa threshold for markedly elevated risk of root resorption. A smaller peak (≈15.6 kPa) was observed at the first-molar apex in the bilateral posterior anchorage group. Such excessive peaks, produced by stress concentrations, also appeared in the FEA by Roscoe et al^29^ under heavy loading (total traction force >225 gf).

The three configurations differed markedly in intrusion efficiency. In the bilateral posterior anchorage group, the mean hydrostatic pressure in the apical PDL was 2.64 kPa in the posterior teeth, which is slightly above the hydrostatic pressure threshold for bone remodelling (2.60 kPa), and only 1.04 kPa in the anterior teeth, which is below this threshold. As a result, effective intrusion occurred primarily in the posterior teeth, while the anterior teeth experienced minimal displacement. In the central incisor-posterior anchorage group, the arch-wide apical mean hydrostatic pressure was 3.35 kPa, placing it within the 2.6 to 4.7 kPa range that supports remodelling but does not reach the accelerated-displacement range. Thus, intrusion was achieved across the arch but with limited efficiency. In the lateral incisor-posterior anchorage group, the arch-wide apical mean hydrostatic pressure reached 5.38 kPa, which falls within the 4.7 to 12.8 kPa accelerated-displacement range. This produced the most efficient intrusion among the three groups, although it came at the cost of the elevated apical hydrostatic pressure peaks noted above.

Although small, localized hydrostatic pressure elevations within the PDL do not necessarily lead to root resorption, hydrostatic pressure peaks of the magnitude observed here still warrant clinical attention. This is most pressing in the central incisor-posterior and lateral incisor-posterior anchorage configurations, where the gain in arch-wide intrusion coverage or efficiency is accompanied by markedly elevated apical hydrostatic pressure (≈40 kPa at the central incisor and ≈35 kPa at the first premolar, respectively); the bilateral posterior anchorage group, whose peak only marginally exceeds the 12.8 kPa threshold, carries a lower but nonnegligible risk at the first molar. When applying forces of this level, clinicians should closely monitor for signs of root resorption and consider adjusting both the magnitude of the applied force and the number and placement of anchorage mini-implants to achieve a more favourable distribution of PDL hydrostatic pressure.

### Additional observations

#### Canine tipping tendency

With the exception of the lateral incisor-posterior anchorage group, the canines showed a tendency toward sagittal crown tipping across all other configurations – an effect also reported by Wang et al.^15^ This behaviour likely stems from the canine’s anatomical position as a transitional pivot between the anterior and posterior segments. Two factors compound at this site: first, the elastic traction from the mini-implants does not pass through the canine’s centre of resistance, generating a tipping moment; second, with anchorage reinforced at both ends of the arch but not at the canine itself, the canine becomes the least-restrained tooth and preferentially yields to the load. The combined result is sagittal tipping rather than true intrusion. This proposed mechanism, however, requires further experimental validation.

#### Asymmetric displacement and hydrostatic pressure between quadrants

In the second quadrant, both tooth displacement and PDL hydrostatic pressure of the posterior teeth were consistently lower than those in the first quadrant. This asymmetry is mainly attributed to the root morphology of the second-quadrant second molar (#27), which has a fused root structure (palatal and mesial root fusion, with a thick distal root), whereas the first-quadrant second molar (#17) has a typical separated, multirooted configuration.

Three interrelated mechanisms account for these findings. First, root fusion and increased thickness increase the effective root-PDL contact area, distributing the applied force over a larger region. This lowers the per-unit-area hydrostatic pressure, thereby reducing hydrostatic pressure elevation at the apex and furcation, which diminishes bone remodelling, while simultaneously enhancing the tooth’s resistance to displacement. Second, the fused and bulkier roots increase the overall stiffness of the tooth-periodontium-bone complex. A thicker root engages more PDL fibres and a larger bone bed, making the anchorage unit mechanically stiffer. Under the same orthodontic force, this stiffer complex undergoes less tipping and translation. Third, the smoother curvature of a fused root, compared with a slender apex, generates a more uniform hydrostatic pressure field along the root surface, blunting localized hydrostatic pressure elevations and making it harder to reach the bone-remodelling threshold.

### Study limitations

Several limitations should be acknowledged. First, the finite element model was constructed from CBCT data of a single patient, which may limit the generalizability of the findings; additional multipatient studies are needed to further validate these results. Second, although a viscoelastic PDL model (third-order Prony series) was used to capture time-dependent mechanical behaviour, the anisotropy of force transmission within the PDL was not considered. This is because human-specific data on PDL fibre orientation and anisotropic properties remain scarce; most existing characterizations are derived from bovine, porcine, or nonhuman primate specimens, and widely accepted constitutive parameters for human PDL anisotropy are still lacking.

## Conclusion

This finite element study systematically evaluated the biomechanical effects of different mini-implant anchorage configurations on maxillary full-arch intrusion with CAs. The main findings are summarized as follows:

1. Unilateral posterior anchorage (buccal or palatal alone) tends to induce coronal tipping of the posterior teeth. In contrast, bilateral synchronous force application effectively balances the buccal and palatal moments, enabling more bodily posterior intrusion.
2. Combined anterior-posterior anchorage establishes a stable two-point force system that facilitates full-arch intrusion with minimal tipping. Among the configurations tested, combined anchorage involving the lateral incisors yields the most favourable outcomes.
3. Adding anterior mini-implant anchorage redistributes the load toward the anterior teeth and improves full-arch intrusion effectiveness; however, it also produces localized hydrostatic pressure elevation at the root apex, thereby increasing the risk of root resorption.
4. Suboptimal vertical control of the canines is observed in most groups, with a tendency toward sagittal tipping, which warrants further investigation.
5. Fused root morphology diminishes the extent of bone remodelling and enhances the tooth’s resistance to displacement.

In summary, this study demonstrates that combined anterior-posterior anchorage with strategically placed mini-implants is an effective strategy for achieving controlled full-arch intrusion with CAs. The findings provide a biomechanical rationale for optimizing anchorage design in clinical orthodontic treatment planning.

## Author contributions

Shengyou Chen: Methodology, software, formal analysis, investigation, writing – original draft, visualization; Shaochuan Feng: Conceptualization, supervision, project administration, writing – review and editing; Wangxi Ni: Methodology, software, formal analysis, investigation; Yutao Pei: Conceptualization, supervision, writing – review and editing; Xiaoyan Wang: Conceptualization, supervision, funding acquisition, writing – review and editing; Xinze Zhang: Conceptualization, resources, supervision, writing – review and editing.

## Ethics statement and consent to participate

This study was approved by the Ethics Committee of Beijing Tiantan Hospital, Capital Medical University, China (KY2024-289-03). Informed consent was obtained from the participant.

## Funding

This research was carried out with the financial support of Beijing Natural Science Foundation (L2510073), Young Elite Scientist Sponsorship Program by Beijing Association for Science and Technology (BAST) (BYESS2024347), Special Program for Clinical Scientists, Beijing Tiantan Hospital, Capital Medical University, China (CS-2025-04).

## Conflict of interest

The authors declare that they have no known competing financial interests or personal relationships that could have appeared to influence the work reported in this article.
